# Telestroke networks for area-wide access to endovascular stroke treatment

**DOI:** 10.1186/s42466-023-00237-9

**Published:** 2023-03-03

**Authors:** Hans Worthmann, S. Winzer, R. Schuppner, C. Gumbinger, J. Barlinn

**Affiliations:** 1 Klinik Für Neurologie, Medizinische Hochschule Hannover, Carl-Neuberg-Straße 1, 30623 Hannover, Germany; 2 Klinik Für Neurologie, Universitätsklinikum Dresden, Dresden, Germany; 3 Klinik Für Neurologie, Universitätsklinikum Heidelberg, Heidelberg, Germany

**Keywords:** Telestroke, Stroke, Teleneurology, Endovascular treatment, Thrombectomy

## Abstract

**Background:**

Endovascular therapy (EVT) offers a highly effective therapy for patients with acute ischemic stroke due to large vessel occlusion. Comprehensive stroke centers (CSC) are required to provide permanent accessibility to EVT. However, when affected patients are not located in the immediate catchment area of a CSC, i.e. in rural or structurally weaker areas, access to EVT is not always ensured.

**Main body:**

Telestroke networks play a crucial role in closing this healthcare coverage gap and thereby support specialized stroke treatment. The aim of this narrative review is to elaborate the concepts for the indication and transfer of EVT candidates via telestroke networks in acute stroke care. The targeted readership includes both comprehensive stroke centers and peripheral hospitals. The review is intended to identify ways to design care beyond those areas with narrow access to stroke unit care to provide the indicated highly effective acute therapies on a region-wide basis. Here, the two different models of care: "mothership" and "drip-and-ship" concerning rates of EVT and its complications as well as outcomes are compared. Decisively, forward-looking new model approaches such as a third model the “flying/driving interentionalists” are introduced and discussed, as far as few clinical trials have investigated these approaches. Diagnostic criteria used by the telestroke networks to enable appropriate patient selection for secondary intrahospital emergency transfers are displayed, which need to meet the criteria in terms of speed, quality and safety.

**Conclusion:**

The few findings from the studies with telestroke networks are neutral for comparison in the drip-and-ship and mothership models. Supporting spoke centres through telestroke networks currently seems to be the best option for offering EVT to a population in structurally weaker regions without direct access to a CSC. Here, it is essential to map the individual reality of care depending on the regional circumstances.

## Background

Neurological diseases are taking on an increasingly important role worldwide. Among neurological diseases, stroke is the disease with the greatest loss of disability-adjusted life years [1].

Since 2015, endovascular treatment (EVT) has been demonstrated as a highly effective therapy for acute ischemic stroke due to large vessel occlusion (LVO) in a narrow time window of up to 6 h after symptom onset [2]. Since expansion of the time window for EVT up to 24 h via patient identification through advanced imaging in 2018, the number of patients who received EVT and benefit from the procedure has largely increased [3, 4].

Primary patient assignment to comprehensive stroke centers (CSC) with all stroke treatment options close to patients’ home would be desirable for all acute stroke patients. However, in a large number of patients long distances to the next EVT-capable stroke center do not allow primary patient referral, especially in more rural or structurally weaker areas. In Germany, in these areas acute stroke treatment is well covered by telestroke networks, as described in a recent analysis displaying the telestroke network landscape. For a more detailed description see review [5]. Telestroke networks were first established two decades ago. The goal is to provide consultation to hospitals in a regional context that participate in emergency stroke care but cannot maintain an on-site neurologist around the clock (spoke centers). The consulting centers are CSCs with certified stroke units and the possibility of EVT.

Due to the described gap in the health care system for EVT in rural areas, this narrative review based on the literature search via PubMed.gov aims to present existing models of care, using the telestroke networks or comparatively direct patient referral to the CSC.

The review clearly focuses on the limited data and concepts in which teleneurological involvement exists. Accordingly, other data is only included if it is important for introductory explanation or context.

## Main text

### Telestroke networks for acute stroke care

In a number of countries including Germany, there is good geographic coverage with stroke units providing acute stroke care. Since complete coverage is not possible, white spots still exist on the map with hospitals that do not have a stand-alone neurology department including a stroke unit and also do not have a neurologist available 24/7. At this point, telestroke networks can ensure access to high-quality therapy according to the stroke unit principle. In some countries, certification of these stroke units as so-called “telestroke units” (telemedicine-supported stroke units) is even possible if all quality principles are adhered to [6].

The increasing establishment of telestroke networks in recent years is based on a principle of spoke centers without a neurology department consulting neurologists in the CSC for support of acute stroke therapy. In a so-called teleconsultation, in spoke centers, stroke patients are to be examined remotely via audio–video consultation by the telestroke-consultant to reliably indicate the acute therapy [7]. At this point, the radiologists and neuroradiologists at the CSC are also involved, without whose cooperation and availability for EVT, the telestroke network could not operate. Cooperations between smaller neurological hospitals without the possibility of EVT can also be integrated into the network structure, for example, if only the CT images are telemedically assessed and a transfer concept between the hospitals exists. Regionality is crucial for a well-functioning stroke network (for more details see review [5]). Current guidelines also address the possibility of optimizing care in underserved areas by connecting to a telestroke network [8].

Good coverage for stroke treatment by telestroke networks in mainly rural areas is shown for Germany in an illustration of the telestroke network landscape including performance figures [5]. There are currently 22 active telestroke networks in Germany, each with at least one stroke unit certified as a CSC. In 2018, teleconsultations were thus performed in a total of 225 hospitals connected via telestroke networks for 27,174 patients diagnosed with stroke. Correspondingly acute treatment in 11% of all stroke patients was supported by teleneurology. The cooperating hospitals (spoke centers) connected to the telestroke networks are mainly hospitals without their own neurology department. But also in other European countries the telestroke service is part of the stroke care concept, e.g. in Spain, which is another example of a country with a high prevalence of telestroke services [9]. In a survey of 43 stroke units, 44% operate telestroke services for spoke centers.

### Intravenous thrombolysis via telestroke networks

First and foremost, telestroke networks are intended to strengthen acute care close to patients’ home, thus enabling rapid access to specialized therapies.

The need of a time-sensitive decision on intravenous thrombolysis (IVT) in patients with acute ischemic stroke in hospitals without the presence of a neurologist was the main reason for establishing telestroke networks. In Germany the reported rate of IVT indicated in telestroke networks was 14.9% [5]. In a statewide established telestroke network in South Carolina, there was a significantly increased IVT rate in hospitals that were connected to the telestroke-network compared with hospitals that were not part of the network [10]. However, in this publication the overall IVT rate was still relatively low at 6.7%.

For comparison of IVT treatment and complication rates, there has been no significant difference between CSCs and spoke centers [11–13]. Even IVT in the extended time window beyond 4.5 h after stroke onset as enabled by patient selection by multimodal imaging using perfusion imaging, was performed in certain spoke centers as shown in an Australian telestroke network [14].

### Endovascular treatment in telestroke networks

In acute ischemic stroke due to LVO, EVT in the anterior and posterior circulation is shown to be a highly effective therapy. This is true for the anterior circulation in a narrow time window up to 6 h after stroke onset using computed tomography angiography (CTA) diagnostics and in a time window of 24 h after patient selection using advanced imaging [2–4]. Recently, the benefit of EVT in basilar artery occlusion has also been demonstrated in a time window of up to 24 h [15, 16].

For stroke care, it would be optimal if all patients could be primarily assigned to an EVT-capable CSC. In reality, however, this is not possible within an acceptable emergency transfer time in rural or structurally weaker areas. On the one hand there would be delays in time-critical recanalization treatment and on the other hand the ambulance service would be bound for an excessively long period by the transport at a time of scarce resources in the health system. Accordingly, treatment access depends on rapid identification of patients in need for secondary transfers, which must be initiated from all hospitals without possibility of EVT comprising the primary stroke centers (PSC) defined as either regional neurological clinics or non-neurological clinics. Within telestroke networks, patients in PSCs, then referred to as spoke centers, can be reliably identified for secondary emergency transfer for either direct EVT or late window advanced perfusion imaging to select patients eligible for EVT. Subsequently, transfer can be rapidly organized with the support of telestroke networks. Thus, these have the task of enabling nationwide coverage by EVT in all patients with acute ischemic stroke due to LVO. Consecutively, the increase of patients with EVT in recent years has also been demonstrated in telestroke networks, e.g. in a large region in the southwestern United States [17].

### Different care models for access of endovascular treatment

The appropriate care model enabling EVT is required to be identified for each region individually, being implemented jointly with the regional ambulance service.

**Fig. 1:**
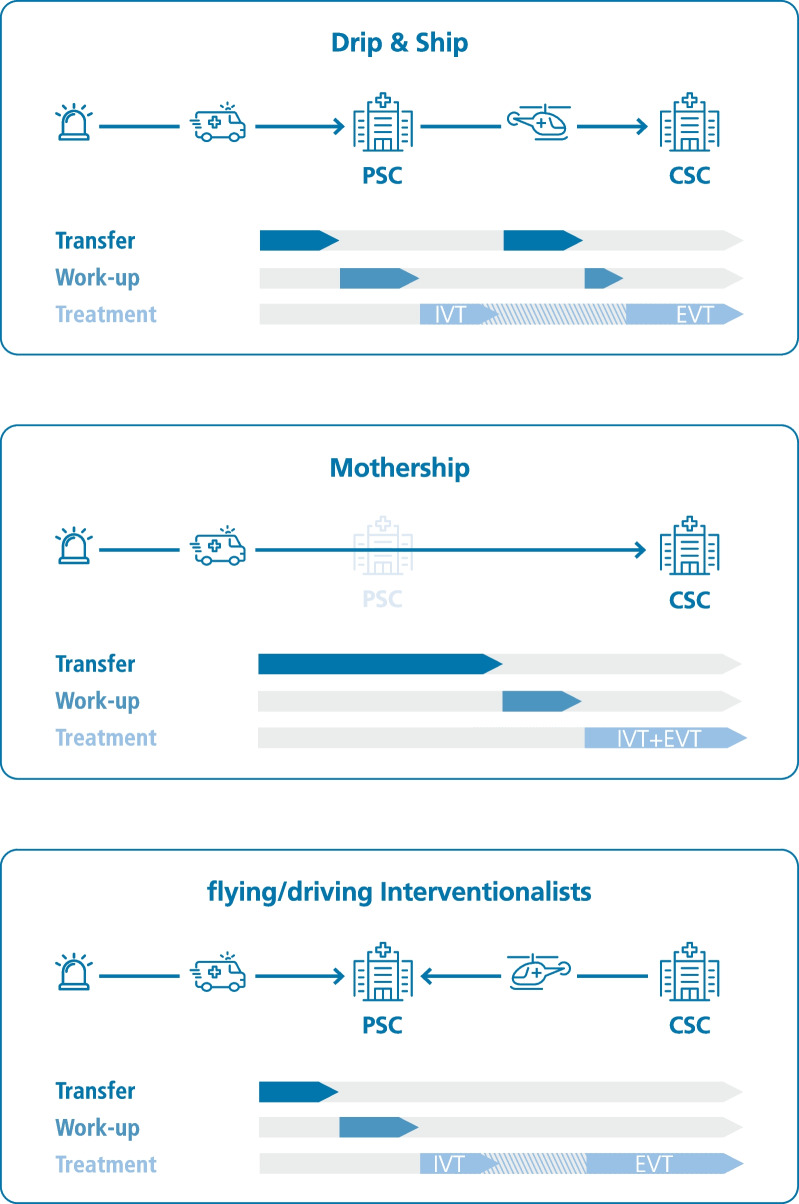
Acute ischemic stroke care models for access to endovascular treatment. *CSC* Comprehensive stroke center; *EVT* Endovascular treatment; *IVT* Intravenous thrombolysis; *PSC* Primary stroke center (without possibility of EVT defined as either regional neurological clinics or non-neurological clinics)

In the mothership model, patients are referred directly to the CSC, which provides continuous care by EVT (Fig. 1). The decisive factor choosing this model should be that the CSC can be reached within a certain time period e.g. 30 min because any further delay would result in a lower rate of IVT and delayed IVT initiation. Besides, CSCs barely can provide capacity for a largely increased amount of stroke patients.

The drip-and-ship principle refers to primary acute treatment including application of IVT in the non-EVT-capable PSC and secondary transfer to the CSC after patient selection for EVT (Fig. 1). In PSCs supported by telestroke networks, then referred to as spoke centers, co-assessment via teleconsultation allows identification not only for IVT but also of potential candidates for EVT. In the current analysis on telestroke networks in Germany, it is shown that 8% of teleneurologically co-assessed patients in the spoke centers undergo secondary emergency transfer for EVT [5].

A third model, and one that has only recently been studied in more detail regarding the use of teleneurology, is the principle of the driving or flying intervention team, according to which the neurointerventionalist located at the CSC drives or flies to the PSC for performing EVT accordingly (Fig. 1). However, this model depends on the provision of personnel resources specifically for the cooperation and the implementation of the required infrastructure at the PSC.

A meta-analysis examined the comparison between the mothership and the drip-and-ship models without teleneurologic support in 7824 patients with acute stroke from 13 studies, most of which were retrospective [18]. The mothership model was associated with a shortened symptom onset to puncture time, a better outcome by mRS at 90 days and fewer bleeding complications. Nevertheless it was pointed out that further prospective randomised trials are needed in order to be able to make statements of adequate quality. Importantly, it has been demonstrated that the onset to treatment times for EVT and the door-in-door-out time have been continuously reduced in recent years for the drip-and-ship model, so the most recent studies possible should be used for evaluation [19]. In addition, the drip-and-ship model could be upgraded by integrating teleneurology, so that a faster indication for the acute therapies IVT and EVT can be made. This has been recently shown in a study with 76 acute stroke patients in New Zealand [20]. Unfortunately, there are only a few studies available that compare the drip-and-ship and mothership models including telestroke consultations. Meta-analyses are not suitable due to the limited data. The available studies are reported below.

The drip-and-ship model has been studied in a telestroke network in Massachusetts. Here, outcomes were studied for 258 EVT candidates with LVO who were secondarily transferred from the spoke centers to the CSC between 2018 and 2020 [21]. In 98 patients who were treated with IVT at the spoke centers functional outcome at discharge and at 3 months was better than patients who were not treated with IVT, while there was no difference in the rate of intracerebral hemorrhage (ICH). It is conceivable that the transfer time can be bridged better when using IVT. Possibly, a complete or partial recanalization was already achieved by IVT in some of the patients. 44% of evaluated patients underwent EVT in the CSC.

In the Stroke telemedicine network in Thuringia (SATELIT), allocation was also based on the drip-and-ship model. All 9937 stroke patients co-assessed by teleconsultations in the period before publication of the large EVT studies in 2015 and after were analysed to identify patients with ischemic stroke due to LVO transferred from the spoke center to the CSC for EVT [22]. Vascular imaging was available more frequently after 2015 resulting in vascular occlusion being diagnosed more frequently at the time of transfer. Accordingly, the indication appears more targeted and secondary patient transfer more effective.

### Patient selection with use of vascular imaging

In a telestroke network in Texas, Reddy et al. studied 400 patients with acute stroke with diagnosed or suspected LVO who were secondarily transferred to the CSC for EVT [23]. Only a minority of patients received vascular diagnostics prior to transfer (17%), so clinical assessment was essential in determining indications. The reasoning for this approach was that the speed of diagnostics is significantly decelerated when CTA was added. Al Kasab and colleagues showed that in 85 transfers of potentially EVT-eligible ischemic stroke patients door-in-door-out (DIDO) times were longer when a CTA had to be added in spoke centers [24]. However, the rate of EVT in the CSC was higher when CTA was performed before transfer. Of note, door-to-groin (DTG) times were not shorter in the CSC when CTA had already been performed. According to the authors, this may have been due to late notification of interventionalists. To support these assumptions, in the SOS-NET from Dresden a team prenotification was implemented resulting in improved treatment times [25].

To be explained, prenotification of the team takes place immediately after the decision to transfer the patient to the CSC, when LVO candidates have already been identified. This should provide instant information to the entire stroke team involved in the process in order to prepare for the patient's arrival. Team prenotification represents an effective measure for the stroke treatment process and can be implemented with manageable effort 8 (for a review see [26]).

In 22 spoke centers in the telestroke network in Massachusetts, a standardized protocol for the CTA process was implemented in 2017 [27]. The protocol addressed CTA indications, acquisition parameters, and image reconstruction method. Previously, transfer was based on clinical suspicion or a high clinical probability of LVO, which was determined based on the National Institutes of Health Stroke Scale, time window, and Alberta Stroke Program Early CT Score. As a result of the introduction of the protocol, there was an increased rate of EVTs performed and correspondingly a higher effectiveness for transfer [27]. Nowadays, in the majority of spoke centers, CTA for suspected LVO is a prerequisite for improved triage for a safely indicated secondary transfer to EVT. This is a crucial condition for reduction of futile transfers to the CSC.

### Comparison of the mothership and the drip-and-ship model using telestroke networks

In the SOS-NET, a comparison was made between the drip-and-ship model and the mothership model in 280 patients with ischemic stroke due to LVO in the anterior cerebral circulation being potentially eligible for EVT during 2015 and 2018 [28]. Of these 163 were treated with EVT. Although there was a greater extent of early infarction signs in the secondarily transferred patients, on the other hand, the DTG time was shorter due to the imaging diagnosis already performed. Importantly, patients’ outcome was comparable between the two models in terms of functional outcome or mortality at 3 months, incidence of symptomatic ICH, and recanalization success (Table 1). Of note there has been an analysis of process times in the SOS-NET, that investigated 48 patients transferred for EVT compared to 103 directly admitted patients at the CSC before the time of the above-mentioned studies [29]. While patients were younger and were more frequently treated with IVT, there was prolonged time from stroke onset to EVT for comparison of the drip-and-ship model and the mothership model. However functional outcome and reperfusion rates were comparable (Table 1).

**Table 1:** Comparison of direct transfer to CSC vs secondary emergency transfer in a telestroke setting in suspected AIS due to LVO

Reference [No.]	Barlinn et al. [29]		Kaminsky et al. [31]		Moustafa et al. [28]	
Number of patients	151		207		163	
Vascular screening criteria	Anterior circulation, LVO		Anterior and vertebrobasilar, suspected LVO		Anterior circulation, LVO	
Center type	CSC/MS	Spoke/DS	CSC/MS	Spoke/DS	CSC/MS	Spoke/DS
Proportion of patients [%]	68.2	31.8	63.8	36.2	55.8	44.2
Age [years]	70 (62–75)*	66 (59–73)*	72.8 (13.5)**	74.5 (15.2)**	76 (65–83)*	76 (66–80)*
NIHSS at admission	15 (11–21)*	15 (12–18)*	16 (10–20) *	17 (13.5–20)*	16 (12–20)*	17 (14–20)*
IVT [%]	**46.6**	**70.8**	**53.8**	**81.3**	59.3	51.4
EVT [%]	100	100	**49.2**	**26.7**	100	100
Onset to groin [min]	225 (175–293)*	319 (270–384)*	200.5 (71.5)**	303.0 (44.3)**	180 (132–220)*	295 (248–340)*
Favorable outcome [mRS 0–2) [%]	Discharge 13.7	Discharge 18.8	3 months 35.1	3 months 32.1	3 months 28.6	3 months 25.4
ICH [%]	11.7 (symptomatic)	4.2 (symptomatic)	**43.3 (all ICH)**	**25.4 (all ICH)**	1.1 (symptomatic)	2.8 (symptomatic)
Mortality [%]	**Discharge 22.6**	**Discharge 8.3**	**3 months 25.2**	**3 months 37.2**	3 months 33.0	3 months 26.8

*AIS* Acute ischemic stroke; *CSC* Comprehensive stroke center; *DS* Drip-and-ship; *EVT* Endovascular treatment; *ICH* Intracerebral hemorrhage; *IVT* Intravenous thrombolysis; *LVO* Large vessel occlusion; *mRS* Modified rankin scale; *MS* Mothership; *NIHSS* National Institutes of Health Stroke Scale; Spoke telemedical site with stroke care

*Median (IQR); **mean (SD); bold statistically significant (*p* < 0.05)

In the same telestroke network, a safety analysis was performed analyzing secondary transfer to the CSC for EVT [30]. No emergency intubation or cardiopulmonary resuscitation was required during transport. Emergency medication was administered in approximately every 10th transfer, with the most common indication being the reduction of excessively elevated blood pressure. The minor medical interventions mentioned above could all be performed by paramedics according to a defined algorithm and did not require the assistance of a physician, which is resource-saving for the transfer.

The application of IVT prior to transfer also does not appear to be associated with increased complication rates or poorer outcomes, as demonstrated in a telestroke network from Massachusetts [21].

A telestroke network in Nancy also prospectively examined the rate of 3-month favorable outcome using the modified Rankin Scale (mRS) in 207 patients assigned to EVT between 2015 and 2017 for comparison of the mothership and drip-and-ship models [31] (Table 1). Spoke centers were at a distance of 36 to 77 miles from the CSC with a mean road transport time of 77 min. Although in spoke centers more patients received IVT, the rate of patients undergoing EVT was lower and they had a longer time interval from symptom onset to treatment compared to CSCs. Outcome at 3 months did not differ for the comparison (Table 1).

There have been few other studies analyzing the differences in the drip-and-ship model compared to the mothership model without explicitly including co-assessment via teleconsultation. In a subanalysis of the DEFUSE 3 trial 182 patients randomized for undergoing EVT or medical therapy alone in an extended time window between 6 and 16 h after symptom onset were divided in patients transferred from the PSC (n = 121) and patients directly admitted to the CSC (n = 61) [32]. Although time intervals from symptom onset to admission in the CSC were longer in transferred patients, functional outcome at 3 months did not differ. However, there is a lack of information to what extent telestroke networks were involved.

In Catalonia, Lopez-Cancio and collegues compared ischemic stroke patients receiving IVT in spoke centers (n = 322) with those patients being treated at PSCs with neurological clinics or CSCs (n = 2897) in a telestroke network from 2013 to 2015 [33]. Patients with ischemic stroke due to LVO were included showing that the rate of EVT was comparable for patients with IVT at spoke centers compared to those with IVT at the PSC or CSC, while the rate was higher when only the subgroup of patients in the CSC was observed.

Importantly, improved patient selection not only increases the rate of EVT, but also better exploits the opportunity for care closer to home and conserves ambulance capacity by avoiding unnecessary transfers. The same study showed similarities in both PSC and CSC with regard to some process times important for stroke care, but also cited longer DNT in the spoke centers, which after all shortened over the observation period and accordingly a learning curve can be assumed. Nevertheless, this aspect draws attention to the fact that adequate quality in telestroke care is not a matter of course, but requires constant optimization by the coordinating team of the network. It is mandatory that the teleconsultant is exempted from other clinical tasks e.g. in the respective emergency departments of the CSC, in order to bring sufficient focus on the teleneurologically treated patient and to enable better process times.

A study in an Australian telestroke network draws attention to the fact that in the drip-and-ship models in spoke centers the targeted process times are only achievable if there is continuous education for the participating physicians [34]. In a system without intensive training for the physicians in the spoke centers, no progress in process times could be achieved, since there was no significant decrease in median door-to-call time over time in the observation period, neither in the overall collective (35 min) nor in the subgroup of patients treated by reperfusion therapy (24.5 min).

### Patient selection with use of clinical scores

A more nuanced picture for comparing the mothership and drip-and-ship models emerges when prehospital decision making can be supported by a score. Thus, in Catalonia, the Rapid Arterial oCclusion Evaluation (RACE) scale has been used with high accuracy to identify stroke patients eligible for EVT [35]. Here, a RACE scale > 4 with a sensitivity of 0.84 and specificity of 0.60 could identify LVO with indication for EVT. In addition, current guidelines and practical guides for acute stroke therapy describe the option of using prehospital scores as potentially useful although the benefit cannot be proven on the basis of current data [8, 36]. In the Medical University of South Carolina (MUSC) Telestroke Program, a comparative study of the different prehospital scores was performed comparing the scores RACE, Field Assessment Stroke Triage for Emergency Destination (FAST-ED), Cincinnati Prehospital Stroke Severity Scale (CPSSS), 3-item stroke scale (3I-SS), and Prehospital Acute Stroke Severity Scale (PASS), and the NIHSS [37]. All scores are in use for decision making for transfer from the spoke center to the CSC. To evaluate the benefit of these scores in intrahospital decision-making, all teleneurologically presented patients from 2014 to 2018 were considered. The authors saw a relatively high error rate in the assignment using the scores, so the development of more appropriate scores is recommended and the eventual assignment must be done without using them for the time being. In addition, especially in remote geographic areas, prehospital scores are not an option for triage, and primary co-assessment by teleconsultation is the only option there, as bypassed transfer directly to the CSCs would mean delaying IVT.

In contrast to the conclusion of the authors of the study, the scores are considered to be usable in a setting with teleneurology in order to pursue a meaningful allocation strategy for at least the majority of patients, which conserves the resources of the local emergency services. However, the scores should be re-evaluated in the local setting in order to be able to improve the concept in a targeted manner e.g. change of the score used or the cut-off used (example of a study in a local setting [38]). A clear recommendation for a single score (one fits all) is currently not made [8]. The decision for or against a score should rather be decided on the basis of regional conditions. Factors that should be considered, among other things, are care levels of the hospitals considered, distance of the hospitals, and use of teleneurological expertise. In addition, regular training of the rescue service is required in order to use the scores sensibly [36].

Instead of using prehospital scores, telemedicine assisted triage of patients already in emergency medical services unit in the rescue vehicle can further accelerate process times and improve patient selection [39]. This was evaluated in a pilot study of 49 patients in the US state of South Carolina. DTG times were shorter in patients who were transferred directly to the CSC for EVT. However, studies in larger patient cohorts are needed for confirmation.

Finally, randomized clinical trials are expected to determine whether there is a difference in outcomes in patients treated with EVT for the comparison of the mothership vs drip-and-ship models. Here, there are ongoing trials PRESTO-F (NCT04121013), TRIAGE-STROKE [40], and the already completed RACECAT Trial [41, 42]. The RACECAT Trial was designed to answer the question of whether outcomes differ significantly in the mothership and drip-and-ship models with respect to mortality, incidence of ICH, and functional outcome as measured by mRS at 90 days. The data revealed no differences in functional outcome or mortality. However, secondary outcomes differed with a higher rate of IVT and a lower rate of EVT in PSCs using the drip-and-ship approach. Of note, 14 of 22 participating PSCs were supported by telestroke-networks, while there was no subgroup analysis with these hospitals.

A retrospective study from the regions participating in the RACECAT Trial showed an increase in the EVT rate between 2016 and 2020 not only in patients assigned according to the mothership model, but also in those assigned according to the drip-and-ship model. In addition, the time interval between arrival at the PSC and groin puncture for EVT decreased [43].

The prehospital setting plays a significant role in transfer to EVT. In addition, a telemedicine approach to optimize transfer for EVT and its process times might be ambulance tracking and a way to communicate via a web-based messenger between the transport team and the CSC neurology team [44].

### Different transportation modes

A small study in an Australian telestroke network with long inter-hospital distances examined modalities for transfers to EVT in 62 patients [45]. The focus was on the comparison of air vs road transfers. Significantly longer DIDO times were found for air transfers, with longer decision-to-departure-time in particular. It also mattered whether patients required intubation prior to transfer. In the very rural telestroke network, it was calculated that the aforementioned delay due to air transfer could only be compensated by the faster air-bound transfer from a distance of approx. 300 km. Automated decision processes could optimize here.

In contrast, a telestroke network in South Carolina and Georgia showed a better outcome in the nearly 50% (n = 100) air borne transferred patients compared to those transferred by ground ambulance (n = 99) [46]. This effect was greater among patients who received EVT due to LVO. A possible confounder discussed was whether the nonrandomized selection for one of the two modes of transport might have been influenced by the fact that patients with airborne transport had less severe co-morbidities. Finally, it seems to depend on the geography of the networks and also on the organization of the ambulance service which means of transport contain the best net benefit.

Certainly, regarding the transportation modes it is equally relevant to investigate whether the approach of a teleneurologically supported Mobile Stroke Unit, which has been shown to be effective for IVT, can also be advantageously applied to EVT for deciding which transfer model to use (for a review see [47]).

### Driving and flying intervention model

Another important model is the more recent principle of the driving or flying intervention team. In this model, EVT is achieved through the deployment of a CSC neurointerventionalist who, after driving or flying to the PSC, performs EVT there. The model requires very close cooperation between the cooperating hospitals, as there is a high level of organizational effort in terms of personnel and infrastructure.

In New York, a CSC Mobile Interventional Stroke Team (MIST) is active to perform EVT for the PSC as quickly as possible. A prospective observational study in 228 patients undergoing EVT found a shorter door to recanalization time when comparing the MIST approach to the drip-and-ship model (Table 2) [48]. The comparison between MIST and mothership was neutral. Complete recovery was achieved in 38% at discharge with MIST, which was more frequent than with the drip-and-ship model at 17%. Regarding long-term outcome, there was only a trend toward better outcome under MIST. In another study in 226 patients, 54% of patients treated with MIST in the early time window (≤ 6 h) and only 28% of patients treated with the drip-and-ship model reached a favorable long-term outcome based on mRS [49]. In the late therapeutic time window (> 6 h), there was no longer a significant effect, so it can be assumed that the time gain due to the accelerated treatment process has an impact on outcome mainly in the early time window, whereas patients receiving EVT in the late time window may benefit less assuming sufficient collateralisation and a preserved penumbra.

**Table 2:** Flying/driving interventionalists concepts with and without a telestroke setting in suspected AIS due to LVO

Reference [No.]	Morey et al. 2020 [48]			Hubert et al. 2022 [53]	
Telestroke setting	Without teleneurology			With teleneurology	
Number of patients	198			157	
Vascular screening criteria	Anterior and vertebrobasilar circulation, LVO			Anterior and vertebrobasilar circulation, LVO	
Center type	CSC/MS	PSC/DS	PSC/MIST	Spoke/DS	Spoke/FIT
Proportion of patients [%]	10.1	57.6	32.3	45.9	54.1
Age [years]	66.8 (± 12.3)**	68.3 (± 14.9)**	69.0 (± 12.8)**	75 (66–79)*	77 (67–82)*
NIHSS at admission	16.6 (± 7.5)**	17.5 (± 6.1)**	15.4 (± 6.1)**	13 (8–18)*	15 (10–18)*
IVT [%]	45.0	45.6	45.3	68	69
EVT [%]	100	100	100	67	83
Initial door to groin [min]	114.3 (± 49.5)**	231.6 (± 102.1)**	144.4 (± 57.7)**; ***p* < 0.05 versus DS**	212 (185–252)*	112 (96–132)*
First imaging to groin [min]	89.8 (± 34.7)**	203.5 (± 91.9)**	126.3 (± 54.4)** ***p* < 0.05 versus DS**	194 (167–226)*	101 (86–115)*
Favorable outcome [mRS 0–2) [%]	(3 months) 40.0	(3 months) 38.9	(3 months) 52.8	(3 months) 39	(3 months) 44
ICH [%]	No data	No data	No data	(Symptomatic) 16	(Symptomatic) 7
Mortality [%]	No data	No data	No data	(3 months) 19	(3 months) 25

It should be noted that further studies on driving interventionalist models exist, but no detailed description regarding the usage of teleneurology has been provided. The very thorough designed study by Morey et al. was included here as an illustrative example

*AIS* Acute ischemic stroke; *CSC* Comprehensive stroke center; *DS* Drip-and-ship model; *EMS* Contact with emergency medical service; *EVT* Endovascular treatment; *FIT* Flying interventionalists model; *ICH* Intracerebral hemorrhage; *IVT* Intravenous thrombolysis; *LVO* Large vessel occlusion; *MIST* Mobile interventional stroke team model; *mRS* Modified rankin scale; *MS* Mothership model; *NIHSS* National Institutes of Health Stroke Scale; Spoke telemedical site with stroke care

*Median (IQR); **mean (SD); bold statistically significant (*p* < 0.05)

Further studies on the driving interventionalist model showed the possibility for acceleration of critical process times for EVT [50, 51]. Although some of the studies provided information that depending on availability teleneurologic co-assessment was partially performed in the PSC, detailed information is lacking [52].

To investigate a similar concept involving teleneurological consultation, a particular approach in patients undergoing EVT was taken through the TEMPIS telestroke network. 157 patients were compared who were either transferred to the CSC by helicopter or treated in the spoke centers by the neurointerventionalist who previously went to the spoke center herself/himself as a "flying interventionalist” (Table 2). A higher EVT rate, earlier treatment initiation saving 90 min, and a trend for better outcome in patients treated by flying interventionalists were achieved. No higher numbers of complications were reported in these patients [53].

So far, an improvement in acute care processes of the driving and flying intervention model has been clearly demonstrated, while the distinct effect on outcomes will be the focus of further investigation.

## Conclusion

Access to EVT in structurally weaker regions without proximity to the CSC is a multifactorial approach that should be considered standard. The primary decision for the mothership model or the drip-and-ship model is made according to the geographical location and access to the CSC including involvement of prehospital scores. The preliminary results of the studies carried out so far are neutral for the comparison of outcomes in the drip-and-ship and in the mothership model. Moreover, the driving or flying intervention team model is gaining increasing attention and will be further explored in studies.

It remains unclear whether the results in certain telestroke networks are transferable to any region, which may differ significantly in process times and infrastructure. Rather, it is important to map the individual reality of care in the best possible way, for which the telestroke networks can form an essential building block.

The support of spoke centers through telestroke networks is a stable solution to provide EVT in a population in structurally weaker regions without direct access to a CSC. The task of the telestroke networks is on the one hand to carry out the teleconsultations in an emergency setting under consideration of vascular cerebral imaging to enable focused patient selection and on the other hand to implement the standards of care and organize a continuous training in the spoke centers to achieve quality-oriented stroke treatment.

## Data Availability

Not applicable.

## Abbreviations

ASCPECTS: Alberta stroke program early CT score
CPSSS: Cincinnati prehospital stroke severity scale (CPSSS)
CSC: Comprehensive stroke center
CTA: Computed tomography angiography
DIDO: Door-in-door-out
DNT: Door-to-needle times
DTG: Door-to-groin
EVT: Endovascular treatment
FAST-ED: Field assessment stroke triage for emergency destination
ICH: Intracranial hemorrhage
IVT: Intravenous thrombolysis
LVO: Large vessel occlusion
MIST: Mobile interventional stroke team
mRS: Modified rankin scale
MUSC: Medical University of South Carolina
NIHSS: National Instititutes of health stroke scale
PASS: Prehospital acute stroke severity scale
PSC: Primary stroke center
RACE: Rapid arterial occlusion evaluation
SATELIT: Stroke telemedicine network in thuringa
SOS-NET: Schlaganfallversorgung in Ost-Sachsen Netzwerk
TEMPIS: Telemedizinisches Projekt zur integrierten Schlaganfallversorgung in der Region Südostbayern
TEMS: Emergency medical services unit
3I-SS: 3-Item stroke scale

## References

[1] GBD 2016 Neurology Collaborators. (2019). Global, regional, and national burden of neurological disorders, 1990–2016: a systematic analysis for the Global Burden of Disease Study 2016. *Lancet Neurology, 18*(5), 459–480. 10.1016/S1474-4422(18)30499-X. 10.1016/S1474-4422(18)30499-X PMC6459001 30879893

[2] Goyal, M., Menon, B. K., van Zwam, W. H., Dippel, D. W., Mitchell, P. J., Demchuk, A. M., Dávalos, A., Majoie, C. B., van der Lugt, A., de Miquel, M. A., Donnan, G. A., Roos, Y. B., Bonafe, A., Jahan, R., Diener, H. C., van den Berg, L. A., Levy, E. I., Berkhemer, O. A., Pereira, V. M., Rempel, J., Millán, M., Davis, S. M., Roy, D., Thornton, J., Román, L. S., Ribó, M., Beumer, D., Stouch, B., Brown, S., Campbell, B. C., van Oostenbrugge, R. J., Saver, J. L., Hill, M. D., Jovin, T. G., & HERMES collaborators. (2016). Endovascular thrombectomy after large-vessel ischaemic stroke: A meta-analysis of individual patient data from five randomised trials. *Lancet,**387*(10029), 1723–1731. 10.1016/S0140-6736(16)00163-X 10.1016/S0140-6736(16)00163-X 26898852

[3] Nogueira, R. G., Jadhav, A. P., Haussen, D. C., Bonafe, A., Budzik, R. F., Bhuva, P., Yavagal, D. R., Ribo, M., Cognard, C., Hanel, R. A., Sila, C. A., Hassan, A. E., Millan, M., Levy, E. I., Mitchell, P., Chen, M., English, J. D., Shah, Q. A., Silver, F. L., Pereira, V. M., Mehta, B. P., Baxter, B. W., Abraham, M. G., Cardona, P., Veznedaroglu, E., Hellinger, F. R., Feng, L., Kirmani, J. F., Lopes, D. K., Jankowitz, B. T., Frankel, M. R., Costalat, V., Vora, N. A., Yoo, A. J., Malik, A. M., Furlan, A. J., Rubiera, M., Aghaebrahim, A., Olivot, J. M., Tekle, W. G., Shields, R., Graves, T., Lewis, R. J., Smith, W. S., Liebeskind, D. S., Saver, J. L., Jovin, T. G., & DAWN Trial Investigators. (2018). Thrombectomy 6 to 24 hours after stroke with a mismatch between deficit and infarct. *The New England Journal of Medicine, 378*(1), 11–21. 10.1056/NEJMoa1706442. 10.1056/NEJMoa1706442 29129157

[4] Albers, G. W., Marks, M. P., Kemp, S., Christensen, S., Tsai, J. P., Ortega-Gutierrez, S., McTaggart, R. A., Torbey, M. T., Kim-Tenser, M., Leslie-Mazwi, T., Sarraj, A., Kasner, S. E., Ansari, S. A., Yeatts, S. D., Hamilton, S., Mlynash, M., Heit, J. J., Zaharchuk, G., Kim, S., Carrozzella, J., Palesch, Y. Y., Demchuk A. M., Bammer, R., Lavori, P. W., Broderick, J. P., Lansberg, M. G., & DEFUSE 3 Investigators. (2018). Thrombectomy for stroke at 6 to 16 hours with selection by perfusion imaging. *The New England Journal of Medicine, 378*(8), 708–718. 10.1056/NEJMoa1713973. 10.1056/NEJMoa1713973 PMC6590673 29364767

[5] Barlinn, J., Winzer, S., Worthmann, H., Urbanek, C., Häusler, K. G., Günther, A., Erdur, H., Görtler, M., Busetto, L., Wojciechowski, C., Schmitt, J., Shah, Y., Büchele, B., Sokolowski, P., Kraya, T., Merkelbach, S., Rosengarten, B., Stangenberg-Gliss, K., Weber, J., Schlachetzki, F., Abu-Mugheisib, M., Petersen, M., Schwartz, A., Palm, F., Jowaed, A., Volbers, B., Zickler, P., Remi, J., Bardutzky, J., Bösel, J., Audebert, H. J., Hubert, G. J., Gumbinger, C. (2021). Telemedizin in der Schlaganfallversorgung—versorgungsrelevant für Deutschland [Telemedicine in stroke-pertinent to stroke care in Germany]. *Nervenarzt, 92*(6), 593–601. 10.1007/s00115-021-01137-6. 10.1007/s00115-021-01137-6 PMC8184549 34046722

[6] Handschu R, Scibor M, Wacker A, Stark DR, Köhrmann M, Erbguth F, Oschmann P, Schwab S, Marquardt L (2014). Feasibility of certified quality management in a comprehensive stroke care network using telemedicine: STENO project. International Journal of Stroke.

[7] Levine SR, Gorman M (1999). Telestroke: The application of telemedicine for stroke. Stroke.

[8] Ringleb, P., Köhrmann, M., Jansen, O., et al. (2023). Akuttherapie des ischämischen Schlaganfalls, S2e-Leitlinie, 2022. In *Deutsche Gesellschaft für Neurologie (Hrsg.), Leitlinien für Diagnostik und Therapie in der Neurologie*. Online: www.dgn.org/leitlinien (accessed at 13 Feb 2023).

[9] Alonso de Leciñana, M., Morales, A., Martínez-Zabaleta, M., Ayo-Martín, Ó., Lizán, L., Castellanos, M., en representación de los investigadores del Proyecto Ictus, GEECV-SEN. (2020). Characteristics of stroke units and stroke teams in Spain in 2018. Pre2Ictus project. *Neurologia (Engl Ed), 8*, S0213–4853. 10.1016/j.nrl.2020.06.012. 10.1016/j.nrleng.2022.03.001 35780047

[10] Simpson AN, Harvey JB, DiLembo SM, Debenham E, Holmstedt CA, Robinson CO, Simpson KN, Almallouhi E, Ford DW (2020). Population health indicators associated with a statewide telestroke program. Telemedicine Journal and E-Health.

[11] Kepplinger J, Barlinn K, Deckert S, Scheibe M, Bodechtel U, Schmitt J (2016). Safety and efficacy of thrombolysis in telestroke: A systematic review and meta-analysis. Neurology.

[12] Waseem H, Salih YA, Burney CP, Abel MA, Riblet N, Kim A, Robbins N (2021). Efficacy and safety of the telestroke drip-and-stay model: A systematic review and meta-analysis. Journal of Stroke and Cerebrovascular Diseases.

[13] Simon S, Forghani M, Abramyuk A, Winzer S, Wojciechowski C, Pallesen L-P, Siepmann T, Reichmann H, Puetz V, Barlinn K, Barlinn J (2021). Intravenous thrombolysis for telestroke in the 3- to 4.5-hour time window. Frontiers in Neurology.

[14] Garcia-Esperon, C., Soderhjelm Dinkelspiel, F., Miteff, F., Gangadharan, S., Wellings, T., O Brien, B., Evans, J., Lillicrap, T., Demeestere, J., Bivard, A., Parsons, M., Levi, C., Spratt, N. J., & Northern NSW Telestroke investigators. (2020). Implementation of multimodal computed tomography in a telestroke network: Five-year experience. *CNS Neuroscience & Therapeutics, 26*(3), 367–373. 10.1111/cns.13224 10.1111/cns.13224 PMC7052799 31568661

[15] Tao, C., Nogueira, R. G., Zhu, Y., Sun, J., Han, H., Yuan, G., Wen, C., Zhou, P., Chen, W., Zeng, G., Li, Y., Ma, Z., Yu, C., Su, J., Zhou, Z., Chen, Z., Liao, G., Sun, Y., Ren, Y., Zhang, H., Chen, J., Yue, X., Xiao, G., Wang, L., Liu, R., Liu, W., Liu, Y., Wang, L., Zhang, C., Liu, T., Song, J., Li, R., Xu, P., Yin, Y., Wang, G., Baxter, B., Qureshi, A. I., Liu, X., Hu, W., & ATTENTION Investigators. (2022). Trial of endovascular treatment of acute basilar-artery occlusion. *The New England Journal of Medicine, 387*(15), 1361–1372. 10.1056/NEJMoa2206317 10.1056/NEJMoa2206317 36239644

[16] Jovin, T. G., Li, C., Wu, L., Wu, C., Chen, J., Jiang, C., Shi, Z., Gao, Z., Song, C., Chen, W., Peng, Y., Yao, C., Wei, M., Li, T., Wei, L., Xiao, G., Yang, H., Ren, M., Duan, J., Liu, X., Yang, Q., Liu, Y., Zhu, Q., Shi, W., Zhu, Q., Li, X., Guo, Z., Yang, Q., Hou, C., Zhao, W., Ma, Q., Zhang, Y., Jiao, L., Zhang, H., Liebeskind, D. S., Liang, H., Jadhav, A. P., Wen, C., Brown, S., Zhu, L., Ye, H., Ribo, M., Chang, M., Song, H., Chen, J., Ji, X., & BAOCHE Investigators. (2022). Trial of Thrombectomy 6 to 24 hours after stroke due to basilar-artery occlusion. *The New England Journal of Medicine, 387*(15), 1373–1384. 10.1056/NEJMoa2207576 10.1056/NEJMoa2207576 36239645

[17] Almallouhi E, Debenham E, Grant C, Spiotta AM, Holmstedt CA, Al KS (2021). Increased telestroke call burden after the extended thrombectomy window trials. Journal of Telemedicine and Telecare.

[18] Mohamed A, Fatima N, Shuaib A, Saqqur M (2022). Comparison of mothership versus drip-and-ship models in treating patients with acute ischemic stroke: A systematic review and meta-analysis. International Journal of Stroke.

[19] Boss EG, Bohmann FO, Misselwitz B, Kaps M, Neumann-Haefelin T, Pfeilschifter W, Kurka N (2021). Quality assurance data for regional drip-and-ship strategies-gearing up the transfer process. Neurol Res Pract.

[20] Scott IM, Manoczki C, Swain AH, Ranjan A, McGovern MG, Shyrell Tyson AL, Hyslop MC, Punter MM, Ranta A (2022). Prehospital telestroke vs paramedic scores to accurately identify stroke reperfusion candidates: A cluster randomized controlled trial. Neurology.

[21] Regenhardt RW, Rosenthal JA, Awad A, Martinez-Gutierrez JC, Nolan NM, McIntyre JA, Whitney C, Alotaibi NM, Dmytriw AA, Vranic JE, Stapleton CJ, Patel AB, Rost NS, Schwamm LH, Leslie-Mazwi TM (2021). 'Drip-and-ship' intravenous thrombolysis and outcomes for large vessel occlusion thrombectomy candidates in a hub-and-spoke telestroke model. Journal of NeuroInterventional Surgery.

[22] Klingner C, Tinschert P, Brodoehl S, Berrouschot J, Witte OW, Günther A, Klingner CM (2020). The effect of endovascular thrombectomy studies on the decision to transfer patients in a telestroke network. Telemedicine Journal and E-Health.

[23] Reddy ST, Savitz SI, Friedman E, Arevalo O, Zhang J, Ankrom C, Trevino A, Wu TC (2020). Patients transferred within a telestroke network for large-vessel occlusion. Journal of Telemedicine and Telecare.

[24] Al Kasab S, Almallouhi E, Harvey J, Turner N, Debenham E, Caudill J, Holmstedt CA, Switzer JA (2019). Door in door out and transportation times in 2 telestroke networks. Neurology Clinical Practice.

[25] Pallesen LP, Winzer S, Hartmann C, Kuhn M, Gerber JC, Theilen H, Hädrich K, Siepmann T, Barlinn K, Rahmig J, Linn J, Barlinn J, Puetz V (2022). Team prenotification reduces procedure times for patients with acute ischemic stroke due to large vessel occlusion who are transferred for endovascular therapy. Frontiers in Neurology.

[26] Kamal N, Smith EE, Jeerakathil T, Hill MD (2018). Thrombolysis: Improving door-to-needle times for ischemic stroke treatment—A narrative review. International Journal of Stroke.

[27] Yu AT, Regenhardt RW, Whitney C, Schwamm LH, Patel AB, Stapleton CJ, Viswanathan A, Hirsch JA, Lev M, Leslie-Mazwi TM (2021). CTA protocols in a telestroke network improve efficiency for both spoke and hub hospitals. AJNR American Journal of Neuroradiology.

[28] Moustafa H, Barlinn K, Prakapenia A, Winzer S, Gerber J, Pallesen LP, Siepmann T, Haedrich K, Wojciechowski C, Reichmann H, Linn J, Puetz V, Barlinn J (2021). Endovascular therapy for anterior circulation large vessel occlusion in telestroke. Journal of Telemedicine and Telecare.

[29] Barlinn J, Gerber J, Barlinn K, Pallesen LP, Siepmann T, Zerna C, Wojciechowski C, Puetz V, von Kummer R, Reichmann H, Linn J, Bodechtel U (2017). Acute endovascular treatment delivery to ischemic stroke patients transferred within a telestroke network: A retrospective observational study. International Journal of Stroke.

[30] Pallesen LP, Winzer S, Barlinn K, Prakapenia A, Siepmann T, Gruener C, Gerber J, Haedrich K, Linn J, Barlinn J, Puetz V (2020). Safety of inter-hospital transfer of patients with acute ischemic stroke for evaluation of endovascular thrombectomy. Science and Reports.

[31] Kaminsky AL, Mione G, Omorou Y, Humbertjean L, Bonnerot M, Lacour JC, Riou-Comte N, Anadani M, Gory B, Richard S (2020). Outcome of patients with large vessel occlusion stroke after first admission in telestroke spoke versus comprehensive stroke center. J Neurointerv Surg.

[32] Sarraj A, Mlynash M, Savitz SI, Heit JJ, Lansberg MG, Marks MP, Albers GW (2019). Outcomes of thrombectomy in transferred patients with ischemic stroke in the late window: A subanalysis from the DEFUSE 3 trial. JAMA Neurology.

[33] López-Cancio, E., Ribó, M., Cardona, P., Serena, J., Purroy, F., Palomeras, E., Aragonès, J. M., Cocho, D., Garcés, M., Puiggròs, E., Soteras, I., Cabanelas, A., Villagrasa, D., Catena, E., Sanjurjo, E., López Claverol, N., Carrión, D., López, M., Abilleira, S., Dávalos, A., Pérez de la Ossa, N., & Catalan Stroke Code and Reperfusion Consortium (Cat-SCR). (2018). Telestroke in Catalonia: increasing thrombolysis rate and avoiding interhospital transfers. *Cerebrovascular Diseases, 46*(1–2), 66–71. 10.1159/000492124 10.1159/000492124 30134222

[34] Kashida, Y. T., Garcia-Esperon, C., Lillicrap, T., Miteff, F., Garcia-Bermejo, P., Gangadharan, S., Chew, B. L. A., O'Brien, W., Evans, J., Alanati, K., Bivard, A., Parsons, M., Majersik, J. J., Spratt, N. J., Levi, C., & Members of Northern NSW Telestroke investigators for this project. (2021). The need for structured strategies to improve stroke care in a rural telestroke network in Northern New South Wales, Australia: an observational study. *Frontiers in Neurology, 12*, 645088. 10.3389/fneur.2021.645088 10.3389/fneur.2021.645088 PMC8064411 33897601

[35] Carrera, D., Gorchs, M., Querol, M., Abilleira, S., Ribó, M., Millán, M., Ramos, A., Cardona, P., Urra, X., Rodríguez-Campello, A., Prats-Sánchez, L., Purroy, F., Serena, J., Cánovas, D., Zaragoza-Brunet, J., Krupinski, J. A., Ustrell, X., Saura, J., García, S., Mora, M. À., Jiménez, X., Dávalos, A., Pérez de la Ossa, N., & Catalan Stroke Code and Reperfusion Consortium (Cat-SCR). (2019). Revalidation of the RACE scale after its regional implementation in Catalonia: a triage tool for large vessel occlusion. *Journal of NeuroInterventional Surgery, 11*(8), 751–756. 10.1136/neurintsurg-2018-014519 10.1136/neurintsurg-2018-014519 30580284

[36] Kobayashi A, Czlonkowska A, Ford GA (2018). European academy of neurology and european stroke organization consensus statement and practical guidance for pre-hospital management of stroke. European Journal of Neurology.

[37] Anadani M, Almallouhi E, Wahlquist AE, Debenham E, Holmstedt CA (2019). The accuracy of large vessel occlusion recognition scales in telestroke setting. Telemedicine Journal and E-Health.

[38] Suzuki Y, Hasegawa Y, Tsumura K, Ueda T, Suzuki K, Sugiyama M, Nozaki H, Kawaguchi S, Nakane M, Nagashima G, Kitamura T, Yokomine K, Sasanuma J (2016). Prehospital triage for endovascular clot removal in acute stroke patients. Acute Medicine & Surgery.

[39] Al Kasab S, Almallouhi E, Grant C, Hewitt D, Hewitt J, Baki M, Sabatino P, Jones D, Holmstedt CA (2021). Telestroke consultation in the emergency medical services unit: a novel approach to improve thrombolysis times. Journal of Stroke and Cerebrovascular Diseases.

[40] Behrndtz A, Johnsen SP, Valentin JB, Gude MF, Blauenfeldt RA, Andersen G, Majoie CB, Fisher M, Simonsen CZ (2020). TRIAGE-STROKE: treatment strategy in acute larGE vessel occlusion: prioritize IV or endovascular treatment-A randomized trial. International Journal of Stroke.

[41] Abilleira S, Pérez de la Ossa N, Jiménez X, Cardona P, Cocho D, Purroy F, Serena J, Román LS, Urra X, Vilaró M, Cortés J, González JA, Chamorro Á, Gallofré M, Jovin T, Molina C, Cobo E, Dávalos A, Ribó M (2019). Transfer to the local stroke center versus direct transfer to endovascular center of acute stroke patients with suspected large vessel occlusion in the Catalan territory (RACECAT): Study protocol of a cluster randomized within a cohort trial. International Journal of Stroke.

[42] Pérez de la Ossa, N., Abilleira, S., Jovin, T. G., García-Tornel, Á., Jimenez, X., Urra, X., Cardona, P., Cocho, D., Purroy, F., Serena, J., San Román Manzanera, L., Vivanco-Hidalgo, R. M., Salvat-Plana, M., Chamorro, A., Gallofré, M., Molina, C. A., Cobo, E., Davalos, A., Ribo, M., & RACECAT Trial Investigators. (2022). Effect of direct transportation to thrombectomy-capable center vs local stroke center on neurological outcomes in patients with suspected large-vessel occlusion stroke in nonurban areas: the RACECAT randomized clinical trial. JAMA*, 327*(18), 1782–1794. 10.1001/jama.2022.4404 10.1001/jama.2022.4404 PMC9073661 35510397

[43] Olivé-Gadea M, Pérez de la Ossa N, Jovin TG, Abilleira S, Jiménez-Fàbrega X, Cardona P, Chamorro Á, Flores A, Silva Y, Purroy F, Martí-Fàbregas J, Rodríguez-Campello A, Zaragoza J, Krupinski J, Canovas D, Gomez-Choco M, Mas N, Palomeras E, Cocho D, Aragones JM, Repullo C, Sanjurjo E, Carrion D, Catena E, Costa X, Almendros MC, Barceló M, Monedero J, Rybyeba M, Diaz G, Ribo M (2022). Evolution of quality indicators in acute stroke during the RACECAT Trial: Impact in the general population. International Journal of Stroke.

[44] Munich SA, Tan LA, Nogueira DM, Keigher KM, Chen M, Crowley RW, Conners JJ, Lopes DK (2017). Mobile real-time tracking of acute stroke patients and instant, secure inter-team communication—The join app. Neurointervention.

[45] Gangadharan S, Lillicrap T, Miteff F, Garcia-Bermejo P, Wellings T, O'Brien B, Evans J, Alanati K, Levi C, Parsons MW, Bivard A, Garcia-Esperon C, Spratt NJ (2020). Air vs. road decision for endovascular clot retrieval in a rural telestroke network. Frontiers in Neurology.

[46] Almallouhi E, Al Kasab S, Nahhas M, Harvey JB, Caudill J, Turner N, Debenham E, Giurgiutiu DV, Leira EC, Switzer JA, Holmstedt CA (2020). Outcomes of interfacility helicopter transportation in acute stroke care. Neurology Clinical Practice.

[47] Navi, B. B., Audebert, H. J., Alexandrov, A. W., Cadilhac, D. A., Grotta, J. C., & PRESTO (Prehospital Stroke Treatment Organization) Writing Group. (2022). Mobile stroke units: evidence, gaps, and next steps. *Stroke, 53*(6), 2103–2113. 10.1161/STROKEAHA.121.037376 10.1161/STROKEAHA.121.037376 35331008

[48] Morey, J. R., Oxley, T. J., Wei, D., Kellner, C. P., Dangayach, N. S., Stein, L., Hom, D., Wheelwright, D., Rubenstein, L., Skliut, M., Shoirah, H., De Leacy, R. A., Singh, I. P., Zhang, X., Persaud, S., Tuhrim, S., Dhamoon, M., Bederson, J., Mocco, J., Fifi, J. T., & Mount Sinai Stroke Investigators*. (2020). Mobile interventional stroke team model improves early outcomes in large vessel occlusion stroke: The NYC MIST trial. *Stroke, 51*(12), 3495–3503. 10.1161/STROKEAHA.120.030248 10.1161/STROKEAHA.120.030248 33131426

[49] Morey JR, Zhang X, Marayati NF, Matsoukas S, Fiano E, Oxley T, Dangayach N, Stein LK, Fara MG, Skliut M, Kellner C, De Leacy R, Mocco J, Tuhrim S, Fifi JT (2021). Mobile interventional stroke teams improve outcomes in the early time window for large vessel occlusion stroke. Stroke.

[50] Seker F, Fiehler J, Möhlenbruch MA, Heimann F, Flottmann F, Ringleb PA, Thomalla G, Steiner T, Kraemer C, Brekenfeld C, Bendszus M (2020). Time metrics to endovascular thrombectomy in 3 triage concepts: a prospective, observational study (NEUROSQUAD). Stroke.

[51] Brekenfeld C, Goebell E, Schmidt H, Henningsen H, Kraemer C, Tebben J, Flottmann F, Thomalla G, Fiehler J (2018). 'Drip-and-drive': Shipping the neurointerventionalist to provide mechanical thrombectomy in primary stroke centers. Journal of NeuroInterventional Surgery.

[52] Osanai T, Ito Y, Ushikoshi S, Aoki T, Kawabori M, Fujiwara K, Ogasawara K, Tokairin K, Maruichi K, Nakayama N, Kazumata K, Ono K, Houkin K (2019). Efficacy of 'drive and retrieve' as a cooperative method for prompt endovascular treatment for acute ischemic stroke. Journal of NeuroInterventional Surgery.

[53] Hubert GJ, Hubert ND, Maegerlein C, Kraus F, Wiestler H, Müller-Barna P, Gerdsmeier-Petz W, Degenhart C, Hohenbichler K, Dietrich D, Witton-Davies T, Regler A, Paternoster L, Leitner M, Zeman F, Koller M, Linker RA, Bath PM, Audebert HJ, Haberl RL (2022). Association between use of a flying intervention team vs patient interhospital transfer and time to endovascular thrombectomy among patients with acute ischemic stroke in nonurban Germany. JAMA.

